# Femoral Hernia

**Published:** 1863-08

**Authors:** Prescott Hewett


Femoral Hernia.—In endeavoring to reduce a hernia I never hesitate to give chloroform, as the immense use which it has proved itself to be, quite overbalances the chance of occurrence of considerable chloroform sickness. In three cases out of four at least the taxis will fail, that is in femoral hernia; it ought not to be prolonged beyond a few minutes, and on no account must force be employed. If you then fail, you must make up your mind to operate, and do so at once. Opening or not opening the sac is quite a matter of secondary importance compared with the period of strangulation, and the more or less forcible taxis. I invariably open the sac, and have never seen any harm arise from this procedure, and if this is not done in some cases the surgeon cannot be quite sure that a bit of bowel is not left in the sac.—(Mr. Prescott Hewett.)—*Id.*

